# Training and Nutrition for Performance: Males, Females, and Gender Differences

**DOI:** 10.3390/nu16233979

**Published:** 2024-11-21

**Authors:** Olga López Torres, Valentín E. Fernández-Elías

**Affiliations:** Faculty of Sports Sciences, European University of Madrid, 28040 Madrid, Spain; valentin.fernandez@universidadeuropea.es

As sports nutrition research evolves, a growing body of evidence highlights the importance of sex-based differences in responses to dietary interventions for athletic performance. In particular, the distinct nutritional needs of female athletes and the impact of chronic energy deficits emerge as central themes. Different studies underscore the complexity of nutritional requirements across genders, emphasizing tailored interventions that can support both performance and long-term health outcomes.

Chronic low energy availability (LEA) is a pervasive issue, particularly among female athletes, which can lead to relative energy deficiency in sport (RED-S) [1]. This condition, marked by insufficient caloric intake to meet energy expenditure, not only impairs athletic performance but also affects bone density, menstrual health, and immune function [2]. The female athlete triad is a serious condition that can lead to health problems [3]. For female athletes in weight-sensitive sports, such as wrestling, maintaining energy balance is crucial yet challenging. In this context, research reveals that female wrestlers often resort to extreme weight-control practices that increase the risk of disordered eating behaviors [4]. The implications of these findings are profound; by focusing on education and structured dietary support, coaches and practitioners can encourage healthy weight management strategies and minimize the risk of nutritional deficiencies and psychological stressors that frequently accompany restrictive eating practices in competitive sports. In addition, adequate protein intake is essential for muscle repair and recovery, particularly following strenuous training [5]. However, sex-specific differences in muscle protein synthesis and amino acid metabolism highlight the need for targeted nutritional interventions [6]. While both male and female athletes benefit from increased protein intake post-exercise, studies indicate that women may require adjusted protein doses to fully support recovery and performance gains, especially in sports with high aerobic and anaerobic demands [7]. Furthermore, the metabolism of branched-chain amino acids (BCAAs) and other essential amino acids is influenced by hormonal factors, which vary significantly across sexes. Research suggests that while amino acid supplementation supports muscle recovery in both male and female athletes, women may require specific attention to amino acid intake to prevent central fatigue and support sustained energy availability, especially in endurance sports [8]. Moreover, the role of amino acids extends beyond immediate muscle repair. Amino acids such as leucine and glutamine play key roles in immune function and energy production, both of which are critical during recovery. Sex differences in the metabolism of these amino acids may affect the efficiency of recovery strategies. While men may metabolize certain amino acids more rapidly, women may benefit from adjusted timing and types of amino acid supplementation to optimize recovery and reduce inflammation [9]. Understanding these nuances in amino acid metabolism offers practical insights into developing personalized nutrition plans that optimize performance while safeguarding health.

However, lipid metabolism also reveals significant insights into recovery, particularly in sports demanding high aerobic capacity [10]. The lipid profile of red blood cells, specifically their glycerophospholipid composition, has been linked to aerobic performance. Lipid-rich cell membranes enhance red blood cell deformability and oxygen transport, directly influencing endurance capacity. Interestingly, female athletes show different lipid utilization patterns than males, which could impact energy availability and recovery. The findings in recent research indicate that optimizing lipid profiles through targeted dietary interventions, potentially with a higher emphasis on omega-3 and omega-6 fatty acids, may help improve endurance and reduce exercise-induced inflammation, thus supporting long-term performance in endurance sports [11].

On the other hand, some supplements can be considered useful for increasing performance [12]. However, not all supplements or ergogenic aids have the same effects in men and women [13]. For instance, creatine supplementation, a well-researched ergogenic aid [14], also appears to impact recovery and muscle strength differently across sexes. While creatine is widely known for its benefits in high-intensity and strength-based activities, studies show that men tend to experience more significant strength gains from creatine supplementation than women. These differences may stem from hormonal variations that influence creatine’s efficacy on muscle strength and mass. For example, men may have a more pronounced response to creatine due to higher basal levels of testosterone, which interacts with creatine to promote muscle synthesis. For female athletes, creatine still provides benefits, but dosages and timing might need adjustment to maximize its effectiveness without leading to excessive water retention or muscle fatigue. The gender-specific outcomes of creatine supplementation underscore the need for tailored approaches that consider the physiological differences in muscular adaptations to strength training [15].

Additionally, hormonal factors contribute to the differential effects of various ergogenic aids and nutrients on muscle strength and endurance across genders. Hormones such as estrogen play a protective role in muscle damage, allowing female athletes to recover faster from intense training, yet this same factor may mitigate some of the hypertrophic responses seen in male athletes [16]. Recent findings suggest that hormonal modulation may play a role in the efficacy of nutritional interventions such as protein and creatine supplementation [7,15]. By considering these hormonal influences, sports nutritionists can create more precise and effective nutritional protocols that account for sex-specific metabolic responses, ultimately aiding in the prevention of overtraining and enhancing recovery. In terms of overall muscle strength, meta-analytical data support the effectiveness of protein and creatine supplementation in strength improvements [16]. However, these effects vary by sex, with men generally experiencing more significant gains in upper and lower body strength compared to women. The reasons for these differences are multifaceted, including variations in muscle fiber composition, hormonal response to resistance training, and differences in baseline creatine storage. These findings advocate for sex-specific supplementation protocols in sports nutrition, where the nuances of muscle physiology are respected to ensure that both male and female athletes can reach their strength potential.

The culmination of these findings underscores the importance of individualized dietary strategies that consider both physiological and psychological factors [17]. Female athletes, particularly in sports that emphasize weight control, require comprehensive support that addresses energy needs, protein intake, and safe weight management strategies [1,3,18]. For male athletes, maximizing gains in strength and endurance through targeted supplementation may be more straightforward but still requires a deep understanding of timing, dosage, and nutrient interactions.

In conclusion, the literature reveals the complexity of training and nutrition interactions influenced by sex-based differences. By integrating these insights into practice, sports nutrition can advance toward more personalized approaches, ultimately promoting optimal performance and health outcomes across diverse athletic populations. Such individualized strategies are not only critical for enhancing individual performance but are also essential for supporting the long-term health and well-being of athletes in high-demand sports environments.
